# Ecological strategies of bacteria shape inherent phage diversity in Atlantic salmon gut microbiomes

**DOI:** 10.1093/ismejo/wraf272

**Published:** 2025-12-08

**Authors:** Caroline S Winther-Have, Jacob A Rasmussen, Xichuan Zhai, Dennis S Nielsen, Thomas Sicheritz-Pontén, Shyam Gopalakrishnan, Martha R J Clokie, Mathias Middelboe, Morten T Limborg

**Affiliations:** Center for Evolutionary Hologenomics, Globe Institute, University of Copenhagen, Copenhagen 1353, Denmark; Center for Evolutionary Hologenomics, Globe Institute, University of Copenhagen, Copenhagen 1353, Denmark; Department of Food Science, Faculty of Science, University of Copenhagen, Rolighedsvej 26, Frederiksberg 1958, Denmark; Department of Food Science, Faculty of Science, University of Copenhagen, Rolighedsvej 26, Frederiksberg 1958, Denmark; Center for Evolutionary Hologenomics, Globe Institute, University of Copenhagen, Copenhagen 1353, Denmark; Centre of Excellence for Omics-Driven Computational Biodiscovery (COMBio), Faculty of Applied Sciences, AIMST University, Bedong, Kedah 08100, Malaysia; Center for Evolutionary Hologenomics, Globe Institute, University of Copenhagen, Copenhagen 1353, Denmark; Becky Mayer Centre for Phage Research, Department of Genetics and Genome Biology, University of Leicester, Leicester LE1 7RH, United Kingdom; Marine Biological Section, Department of Biology, University of Copenhagen, Strandpromenaden 5, Helsingør 3000, Denmark; HADAL & Nordcee, Department of Biology, University of Southern Denmark, Campusvej 55, Odense 5230, Denmark; Center for Evolutionary Hologenomics, Globe Institute, University of Copenhagen, Copenhagen 1353, Denmark

**Keywords:** phage diversity, virus ecology, *Aliivibrio*, *Mycoplasma*, *Salmo salar*, microbiota dysbiosis, virome, intracellular bacteria, microbial ecology

## Abstract

Understanding host-specific phage diversity is essential for deciphering the complex dynamics shaping microbial ecology and evolution. However, the lack of inherent host associations between uncultivated bacteria and their viruses remains a major limitation to understanding the drivers of viral diversity and its role in bacterial ecology, particularly given the intricate specificity of phage-host interactions. The naturally low complexity of the gut microbiota within piscivorous fish, such as Atlantic salmon (*Salmo salar*), makes it a valuable model for unravelling ecological patterns of viral diversity in the context of a limited bacterial species composition, and to explore the impact of an invading pathogen on the ‘steady-state’ viral community. The intestinal microbiota of the salmon studied here, was in some cases dominated by a salmon-associated *Mycoplasma* or increasing levels of an opportunistic *Aliivibrio,* the latter observed in response to a disease outbreak. The two bacteria are distinctively different in their ecological strategies and their overall genomic and functional properties. A pronounced difference was observed in the gut viral communities and diversity, depending on whether it was dominated by a commensal or an invading bacterial species. Samples dominated by *Mycoplasma* sp. had few to no viruses, whereas samples dominated by *Aliivibrio* sp. had viral communities comprising up to 22 viral taxonomic operational units. This study provides unique insights into the significance of bacterial ecological trade-offs linked to niche adaptation and how these affect the associated viral communities in a natural host-controlled environment.

## Graphical Abstract

**Graphical Abstract:**
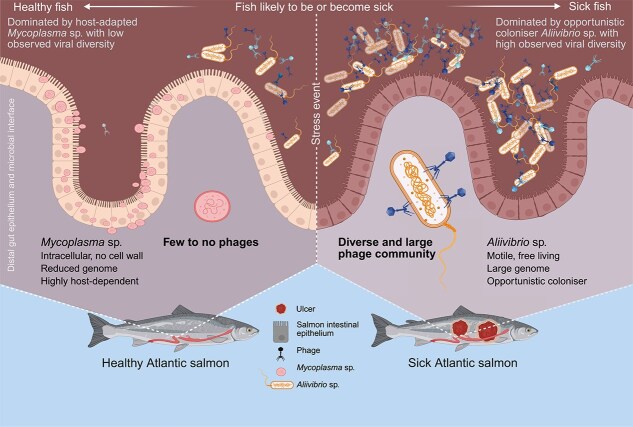

## Introduction

Due to their host specificity, bacteriophage diversity is inherently linked to their host community composition. Conservative estimates predict 10–100 phage species per bacterial species [1], and phage abundance on average exceeds bacterial cells by ~10-fold [2]. Phages significantly influence the mortality, community composition, and evolution of their bacterial hosts through predation and gene transfer, and can regulate host metabolic processes through phage-encoded auxiliary metabolic genes [3–5]. Bacterial diversity is considered a key driver of phage diversity, with more diverse bacterial communities supporting greater phage richness [6]. However, the role of bacterial ecological properties beyond species richness in shaping phage communities remains poorly understood. Despite the importance of understanding these dynamics, elucidating the drivers of species-specific phage diversity remains a major challenge. With the advances in high throughput sequencing and bioinformatic tools, a tremendous number and diversity of viruses are routinely uncovered [7], contributing novel insights into their function and distribution. However, the ability to link these, often novel and uncharacterised viruses to their correct host remains challenging despite its importance for understanding the interplay between viral composition and diversity and their bacterial host community [8].

One approach to addressing these challenges in natural systems is to study a low-diversity environment with a well-characterised bacterial community [9–11], that allows identification of phages associated with specific hosts. Atlantic salmon (*S. salar*), valued for its ecological and aquacultural importance, is an interesting candidate, as it has become a well-established model organism with multiple studies contributing to mapping of the gut microbiota and resolving its impact on salmon health [10, 12–14]. A large proportion of the bacteria found in Atlantic salmon appears to be transient [15]. Nevertheless, one species of *Mycoplasma* is consistently found and considered to have codiverged with wild Atlantic salmon, where it has a stabilising effect on the immune system and micronutrient metabolism [10, 15]. However, stress or disease outbreaks cause marked changes in the gut microbiota [16–18]. In some salmonids these changes have resulted in an increase in bacterial diversity [19], whereas in other systems, the displacement of the resident *Mycoplasma* by opportunistic *Aliivibrio* sp. has been reported [16, 18, 20]. The virome of Atlantic salmon has also been investigated, focusing on the eukaryotic salmon-associated viruses in an effort to identify putative viral pathogens [21, 22]. However, the diversity and dynamics of bacteriophage communities in the intestinal environment of Atlantic salmon or other teleost species during stable and disease-associated conditions have not previously been reported. Leveraging the Atlantic salmon as a natural model, we tested the hypothesis that inherent phage diversity is shaped by the contrasting ecological strategies of intestinal bacteria including opportunistic colonisers and host-adapted bacteria within the gut microbiome.

Metagenomics provides critical insights into the dynamics of uncultivated bacteria and their associated viruses [23]. However, capturing viral sequences can be challenging in host-associated environments with low microbial biomass. To address this, we concentrated viral particles from Atlantic salmon gut contents to prepare virus-enriched metagenomes. This approach allowed us to explore how viral communities were affected by changes in the bacterial microbiota during disease outbreaks. The gut microbiota was dominated by two distinct bacterial genera representing contrasting ecological strategies: the salmon-associated *Mollicute Mycoplasma* sp., a host-adapted symbiont in healthy fish and the opportunistic Gammaproteobacterial coloniser *Aliivibrio* sp. associated with diseased fish. The contrast between these two bacteria and their disparate ecological strategies, ranging from highly host-dependent to opportunistic coloniser, enabled us to study host-specific drivers of viral communities in a natural *in vivo* environment without reducing true complexity. Our results highlight the value of simple, natural microbiota systems to unravel dynamics of the species-specific virus-bacteria interactions and highlight the importance of understanding the viral component of microbial ecosystems and how microbiome changes affect viral community composition.

## Materials and methods

### Study design and background

In May 2019, distal gut content samples were collected from juvenile Atlantic salmon (*S. salar*) reared in a flow-through system at the LetSea land facility in Bjørn, Norway, as previously described [16]. The fish were acclimatised in brackish water (24 ppt, 12°C) for 53 days, then transferred to UV-treated seawater (33–34 ppt) in 12 tanks containing 200–300 fish each. Prior to transfer, water temperature was raised by 3°C to support growth. Following transfer, a spontaneous outbreak of tenacibaculosis caused by *Tenacibaculum dicentrarchi* occurred [16], resulting in large skin ulcers in some fish. Due to the contained tank environment, all fish were exposed to the pathogen and expected to eventually develop disease symptoms. However, at the time of sampling, both phenotypically healthy and visibly sick salmon co-occurred presenting a unique opportunity to compare relatively more resilient individuals with less resilient ones.

All salmon included in this study were sampled at the same time point during the progressing disease outbreak. Only those with visible disease signs were classified as ‘sick,’ whereas unaffected, seemingly resilient fish were labelled ‘healthy.’ This classification system enabled comparison of how disease-driven host phenotype relates to gut bacterial community composition and associated viral communities. A 16S rRNA gene amplicon analysis of the samples revealed a simple gut microbiota, dominated by two species: *Aliivibrio* sp. and *Mycoplasma* sp. [16]. Their relative abundance correlated with health status, sick fish showed higher *Aliivibrio* sp. abundance, whereas healthy fish had more *Mycoplasma* sp. [16].

**Figure 1:**
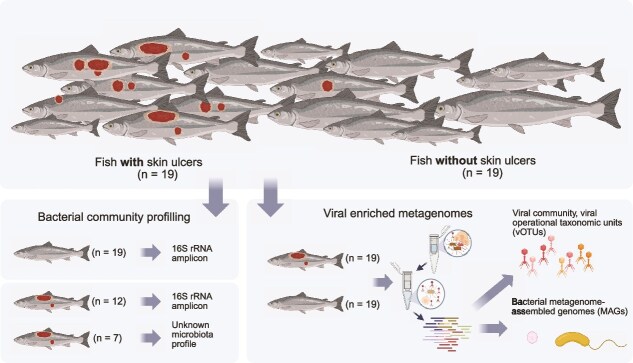
Study setup of the 38 juvenile farmed Atlantic salmon, of which gut content was collected and processed to collect 16S rRNA gene amplification (cf. [16]) and virus-enriched metagenome data (this study). Figure created with BioRender.com.

To investigate viral communities in this simple natural host system, we selected 19 samples from each salmon phenotype (healthy and sick). Since seven samples from sick fish in the original study [16] were depleted, we substituted seven samples from other salmon labelled as sick, but that was not included in the original 16S rRNA gene study (Fig. 1). Selected replacement fish had similar size and Fulton’s condition factor as the depleted samples. These replacement samples were expected to represent the *Aliivibrio* sp.-dominated gut microbiota profile characteristic, as previous work demonstrated that phenotypically sick fish tended to have an *Aliivibrio* sp.-dominated microbiota [16]. However, to maintain analytic rigour, these seven samples were labelled as having ‘unknown microbiota profile’ in downstream analyses, serving validation purposes while balancing the expected distribution of bacterial community profiles among all 38 samples.

### Virome extraction and sequencing

The viral metagenomes from the 38 fish samples were recovered using a previously published protocol for extraction and library preparation [24]. Briefly, DNA/RNA from 500 μL of thawed distal gut content samples was centrifuged and the supernatant was filtered through a PES 0.2 μm 33 mm syringe to limit cellular DNA and allowing DNA extraction of viral particles in the filtrate. The nucleic acids from the filtrate were extracted using QIAmp Viral RNA Mini Kit (Qiagen, Hilden, Germany). The extracted nucleic acids were amplified using short multiple displacement amplification (sMDA) using the GenomePhi V3 kit (GE Healthcare Life Science, Marlborough, MA, USA) with shortened amplification time (30 min) to include single-stranded DNA viruses. To include potential RNA viruses, reverse transcription (RT) was achieved following the kit and protocol by SuperScript IV VILOTM Master Mix Invitrogen (ThermoFisher Scientific, Inc., Waltham, MA, USA). Finally, libraries were constructed and indexed using the Illumina Nextera XT library preparation kit guidelines (FC-131-1096). Samples were pooled based on DNA concentration measures using Invitrogen Qubit, to a final volume of 20 μL. The libraries were analysed by Fragment Analyzer (BioLabTech, Ltd, Ukraine), and sequenced on a NovaSeq 6000 System (Illumina) as paired-end 2x150 bp reads.

### Bacterial profile of 16S rRNA gene data

The microbiota composition based on 16S rRNA gene amplicon and quantitative PCR (qPCR) data was generated and described in detail previously [16]. Data from samples overlapping with this study, were downloaded from the publicly available github page (https://github.com/DavideBozzi/Bozzi_et_al_2020_analysis).

### Read processing and assembly

The raw sequencing data underwent a two-step processing using BBtools (bbduk.sh) v.39.01 [25]. Adapter sequences and phi-X174 reads were removed with parameters (ref = adapters,phix k = 23 hdist = 1 tpe tbo), followed by quality trimming (qtrim = rl trimq = 12 minlength = 51 maxns = 2 maq = 3). Eukaryotic reads were removed with extract_kraken_readsbb.py script using Kraken2 NCBI RefSeq V205 database [26]. Following the quality control and host removal, the samples were assembled using SPAdes v3.15.5 [27] (−-meta --only-assembler -k 25,33,55,77,95,101,127) both as individual assemblies and a combined assembly of the virome libraries, aiming to enhance the overall data yield. The quality of the assemblies was assessed using Quast v5.2.0 [28].

### Virus identification and profiling

Viral contigs from both assemblies were predicted and annotated by geNomad v1.7.4. [29] using default settings. Only viral contigs larger than 3 kb and with a calibrated virus score of minimum 0.8 were used for downstream analysis. The quality of the predicted viral contigs was estimated using CheckV v1.0.1. [30], and manually curated. The predicted viral contigs from all the assemblies were merged and de-replicated into viral operational taxonomic units (vOTUs) at 95% average nucleotide identity (ANI) across 85% of the alignment fraction as according to community guidelines [8] using the CheckV anicalc.py and aniclust.py scripts [30], with the longest contig of each cluster selected as the representative.

To estimate the coverage of vOTUs across samples, reads from the virus-enriched metagenomes were mapped to the vOTU sequences using Bowtie2 v2.5.4 [31]. The resulting SAM files were converted into BAM files and sorted using Samtools v1.21 [32]. CoverM v0.7.0 was used to generate coverage statistics from the sorted BAM files to calculate RPKM (reads per kilobase per million) value of each vOTU, after normalisation by sequencing depth (per million reads) and contig length (in kilobases), for the individual contigs across samples [33]. Due to the sequencing depth, a lenient cutoff of 2% covered fraction was used for the final vOTU table.

To group annotated genes by function, Pharokka v1.5.0 with the -g prodigal-gv flag was used to ensure compatibility with alternative stop codon usage [34, 35].

To predict bacterial hosts based on the vOTU genomes, iPHoP v1.3.2 was used with default settings [36].

### Genome-resolved metagenomics

We applied genome-resolved metagenomics to gain genomic and functional insights into the bacterial communities inhabiting the Atlantic salmon gut. Due to the low microbial biomass in the gut contents, both salmon and bacterial DNA were inadvertently recovered in the filtrate and subsequently sequenced, enabling the recovery of metagenome-assembled genomes (MAGs) from the co-assemblies. MAG recovery and analysis were performed using the anvi’o programs and platform v8.0 [37, 38]. Briefly, a contig database was constructed with open reading frames predicted by Prodigal [39]. HMMER v3.3 [40] identified single-copy core genes for assessment of genome completeness and contamination. Predicted genes were annotated using NCBI’s Clusters of Orthologous Groups COG2021 [41] and the KOfam HMM database of KEGG orthologs (downloaded 6 October 2023) [42, 43]. To generate MAGs, we mapped sequencing reads to the annotated contigs and clustered the resulting coverage profiles to identify single-copy variants. Contigs were initially binned into MAGs using Metabat2 v2.15 [44], followed by manual refinement in the anvi’o platform. We retained only bins with completion scores exceeding 50% and redundancy below 5%, in accordance with MIUVIG guidelines for medium and high-quality MAGs [45]. For phylogenomic analysis and placement, we estimated initial taxonomic classification based on single-copy core genes using GTDB v214 [46]. We then retrieved publicly available genomes from RefSeq (last accessed November 2023) [47], for the two genera corresponding to the dominant bacteria identified with 16S rRNA gene analysis and the only two MAGs recovered: *Mycoplasma* sp. and *Aliivibrio* sp. For the *Mycoplasma*, only fish-associated genomes were used [10, 48], whereas for *Aliivibrio*, we included all publicly available genomes from Refseq (Supplementary information) to construct a phylogenetic tree. The tree was generated by concatenating and aligning amino acid sequences of single-copy core ribosomal proteins, then visualised using FastTree v2.1 [49]. For comparative genomics of *Aliivibrio* genomes, we selected the closest relatives to our study-specific MAG (Fig. S1). To assess genome similarity within the two pangenomes, we calculated ANI and evaluated shared and unique genomic features. We reconstructed metabolic pathways for all genomes used in the pangenomic analysis and calculated the completeness of KEGG modules [43] based on KEGG annotations. Finally, we predicted phage defence systems using PADLOC v2.0 [50], and screened for antimicrobial resistance and virulence genes using abricate v1.0.1 [51].

### Statistical analysis and visualisation

Microbial and viral composition was analysed in Rstudio v4.4.1. [52], using R packages including tidyverse (dplyr, tidyr, readr) [53], phyloseq for microbiome object management [54], hilldiv for normalisation of relative abundance and diversity estimation [55], and ggplot2 packages for visualisation [56]. Statistical analyses employed base R stats package, psych for correlation tests [57], rstatix for post-hoc comparisons [58], and MASS for data manipulation [59]. Specific methods included total sum scaling (TSS) normalisation, Wilcoxon rank-sum tests, Pearson correlations with false discovery rate adjustments, Shapiro–Wilk normality tests, Tukey HSD and Dunn’s tests for pairwise comparisons. Data were transformed and reshaped using reshape2 [60], with gridExtra [61] used for plot arrangement, enabling comprehensive microbiome and metabolic module completion analysis.

To assess potential confounding effects of fish health status on bacterial-phage relationships, marginal and conditional correlations were computed between dichotomised bacterial profile and phage presence variables. Bayesian models were implemented using the *rethinking* package [62] to evaluate the effects of health status and bacterial profile (Fig. S2). Phage presence was modelled as $V\sim Bernoulli(p); logit(p)=$  $\alpha$+${\beta}_BB$+${\beta}_HH$, where V represents phage presence/absence, B denotes bacterial grouping (*Aliivibrio* sp. or *Mycoplasma* sp.), and H indicates health status. Additionally, viral diversity*—*measured as the number of unique viral OTUs detected—in phage-positive samples were modelled as ${N}_V\sim Poisson\left(\lambda \right)\!;\, \mathit{\log}\left(\lambda \right)=\beta \left[B\right]$, where ${N}_V$ represents vOTU counts and $\beta \left[B\right]$ varies by bacterial profile. In both these models, all the coefficients had weakly informative normal priors.

## Results

### Virome data reveal low phage numbers within the Atlantic salmon intestinal environment

Shotgun sequencing of virus-enriched metagenomes from 38 juvenile salmon distal gut samples yielded 168 million reads. Kmer-based taxonomic profiling revealed that the vast majority of reads 96.2% (SD ± 3.5%) were of salmon origin, with bacterial reads comprising 0.9% (SD ± 2.31%) and viral reads only 0.04% (SD ± 0.11%) across samples. This high abundance of Atlantic salmon reads is expected based on previous studies using bulk metagenomes to explore the intestinal microbiota in salmon [48, 63]. The recovery of viral reads is also similar to findings from other studies using viral enrichment techniques [64].

After removal of salmon reads, the co-assembly yielded 18 307 contigs whereas the single sample assemblies had an average of 837 contigs (SD ± 1171.5) with the lowest recovery being 100 contigs and the highest being 4885 contigs from a single sample. Of the 18 307 contigs, 198 contigs were predicted to be viral. Following clustering and de-replication of contigs from both co-assembly and individual assemblies, 93 nonredundant vOTUs were obtained, of which 22 passed quality control with a weighted average length of 13 541 bp. Of the 22 vOTUs, 21 were classified as belonging to the dsDNA class Caudoviricetes and one as unclassified. No ssDNA or RNA viral genomes were recovered, despite using sMDA and RT. sMDA was appropriate, as it converts ssDNA to dsDNA and preferentially amplifies small, circular ssDNA genomes, whereas RT enables RNA virus detection [65]. The absence of these viral genomes, despite the use of targeted methods, suggests low abundance of ssDNA and RNA viruses in the gut content.

### Metagenome-resolved metagenomes and pangenomic analysis reveal a bacterial composition dominated by salmon-associated *Mycoplasma* and *Aliivibrio* species

The 16S rRNA gene amplicon analysis was used to estimate bacterial community composition and identified the two dominant bacteria to belong to the genera *Mycoplasma* and *Aliivibrio*, respectively [16]. However, due to the uncharacterised status of these bacteria and the inherent limitations of 16S rRNA gene analysis, their precise taxonomic placement and genomic features remained unknown. MAGs are commonly retrieved from co-assemblies as they enable recovery of even low abundance bacterial species, which we leveraged to improve phylogenetic understanding and gain genomic insights of the bacteria. We used the bacterial read proportion of the assembled contigs from all 38 samples to recover MAGs from the virus-enriched metagenomes.

Consistent with the 16S rRNA gene findings, the MAGs confirmed a very low diversity bacterial community in the salmon gut. A total of three MAGs were recovered, *Aliivibrio* sp. (98.6% completion and 2.8% redundancy), *Mycoplasma* sp. (67.6% completion and 1.4% redundancy) and a *Pseudomonas* sp. (60% completion, 11.3% redundancy). For the remainder of the paper, we focus on the two dominant *Aliivibrio* and *Mycoplasma* MAGs, as the *Pseudomonas* MAG, though successfully recovered, was a low-quality draft, had a read coverage of 0.04% and was not detected in the samples of which 16S rRNA gene amplicon data was recovered.

**Figure 2:**
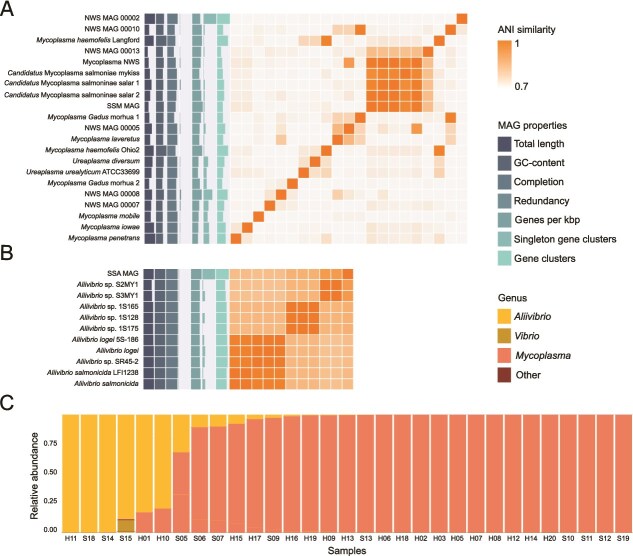
Comparative metagenomics and abundance profiling across *Mycoplasma* and *Aliivibrio* species. (A) Heatmap of ANI across *Mycoplasma* genomes selected based on previous work on salmonid-related *Mycoplasma* [9]. Barplots on y-axis represent (from left to right) total length, GC content, completion, redundancy, number of genes per kb, single gene clusters, and number of gene clusters for *Mycoplasma* genomes in the comparison. (B) Heatmap of ANI across *Aliivibrio* genomes with barplots showing genome properties as in (A). Selection of *Aliivibrio* genomes was carried out based on prior phylogenetic comparison (Supplementary information). For Figures A and B, genomes obtained in this study are labelled as SSM MAG and SSA MAG, respectively. (C) Bar plots showing relative abundance of SSA and SSM across salmon samples based on 16S rRNA gene counts. Fish ID starting with H or S are from salmon with healthy or sick phenotype, respectively. Samples are ordered by bacterial profile to emphasise microbial composition and diversity across samples, independent of health status, as the subsequent analyses are based on microbiota grouping and not the scored fish disease state. Low abundant taxa, except *Vibrio* that was abundant in sample S15, were classified as other for illustrative purposes.

To assess the phylogenetic relationship of the MAGs and extend their taxonomic placement beyond genus, we retrieved fish-associated bacterial genomes from both the *Mycoplasma* and *Aliivibrio* genera and conducted a pangenomic analysis. The pangenomic analysis revealed the *Mycoplasma* sp. MAG to be the same species as the previously isolated *Candidatus* Mycoplasma salmoninae salar from both farmed and wild Atlantic salmon [10], sharing 98.8% ANI (Fig. 2A). For the remainder of this paper, we refer to this study-specific *Mycoplasma* MAG as SSM. The *Aliivibrio* sp. did not match any genomes at the species level, but clustered closely to two isolates, *Aliivibrio* sp. S2MY1 and S3MY1 (Fig. S2) with a 90% ANI similarity (Fig. 2B). *Aliivibrio* sp. S2MY1 and S3MY1 were both identified as colonising and largely undescribed species of *Aliivibrio*, isolated from the gastrointestinal mucosa from thermally stressed, sea cage reared Atlantic salmon in south-east Tasmania [18]. Similar to the SSM, here we refer to the *Aliivibrio* MAG as study-specific *Aliivibrio* SSA.

The bacterial community composition was estimated based on relative abundance across samples. We used the 16S rRNA gene amplicon data for the initial grouping of samples instead of MAG coverage, despite high concordance (Fig. S3), due to higher coverage. Of the 31 samples with a 16S rRNA gene profile the relative abundance was dominated by SSM (*n =* 21) or SSA (*n =* 9) (Fig. 2C). The 31 samples with a known microbiota profile, were grouped according to the relative contribution of SSM. SSM dominance of ≥90%, was treated as a healthy fish baseline, as the bacteria is associated with healthy and wild salmon [10, 16], whereas less contribution of SSM in the fish microbiota indicated a commencing SSA colonisation of the gut, associated with the tenacibaculosis outbreak and a dysbiotic gastric environment. The seven new samples lacking 16S rRNA gene microbiota profiles (Sample ID S01, S03, S04, S08, S16, S17, S20) were grouped separately.

### Viral communities are significantly different depending on the dominant bacteria

**Figure 3:**
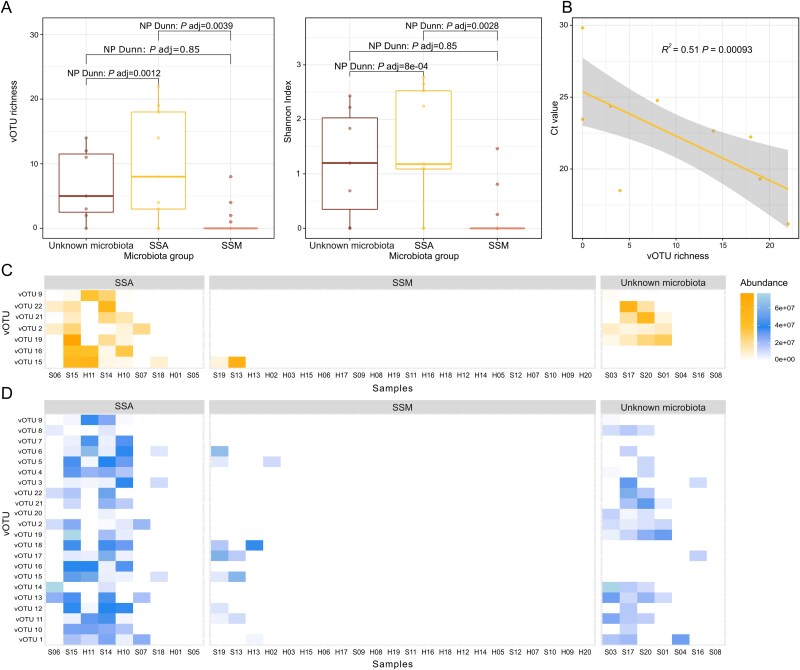
Overview of the viral community distribution across samples. (A) Diversity indices for the vOTUs using observed and Shannon index. Probability testing with NP Dunn statistics, show a significant difference in viral diversity between communities associated with SSA (*Aliivibrio* sp.) or SSM (*Mycoplasma* sp.). The viral communities from samples with an unknown microbiota profile show the same patterns as SSA dominated samples. (B) Regression analysis between Ct values (inverse proxy for bacterial biomass) and the vOTU richness recovered from samples in the SSA group. (C) Heatmap of the viral richness across samples. Samples are ordered based on Bray–Curtis dissimilarity index for all vOTUs and faceted bacterial groupings. Fish ID starting with H or S are from salmon with healthy or sick phenotype, respectively. Similarly, the bars above each plot group samples based on whether the gut microbiota is dominated by SSA or by SSM. (D) Same as (C) but filtered for vOTUs predicted to infect *Vibrionaceae*.

The viral community analysis had distinct patterns in terms of distribution and host associations. The 22 vOTUs that were recovered had a clear distribution based on their associated bacterial grouping, and due to the low diversity of the system we were able to correlate the vOTUs to a single bacterial host species. Viral diversity differed significantly between SSA and SSM dominated samples (Fig. 3A). This diversity was unrelated to overall bacterial load of the samples, with SSA samples exhibiting higher viral diversity despite their lower bacterial density compared to SSM-dominant samples (Fig. S4). Although no significant relationship was observed between viral diversity and overall bacterial load across sampled fish, vOTU community diversity correlated negatively with SSA-associated cycle threshold (Ct) values (Fig. 3B). This inverse relationship demonstrates that phage richness increases with SSA abundance, suggesting the viral diversity expands in response to greater host availability.

Bayesian modelling confirmed these observational patterns, showing that both bacterial profile and health status were associated with phage detection. SSA-dominated fish were more likely to harbour phages than those with SSM profiles, whereas sick fish demonstrated higher phage detection rates than healthy individuals within both bacterial communities (Fig. S5 and S6).

Host predictions identified seven vOTUs predicted to infect the genera *Vibrionaceae*, of which four were predicted to specifically infect *Aliivibrio* genus (Fig. 3D). The remaining 15 vOTUs had no host predictions. Their association, however, indicates that the majority of the 15 vOTUs could be SSA phages, as they strongly correlated to the presence of SSA in the samples (Fig. 3C). The vOTUs were not assigned taxonomy further than class level, and their lack of host assignment to *Aliivibrio* species likely reflects the limited genomic data available for this species.

**Figure 4:**
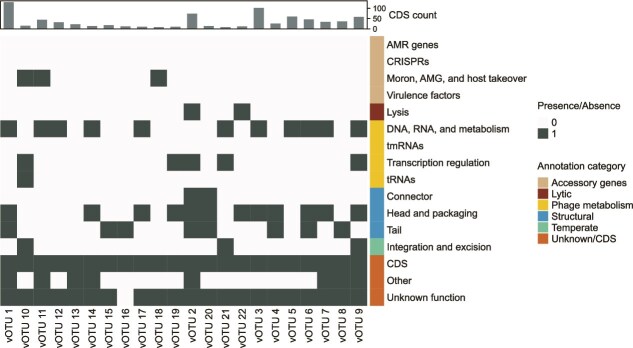
Presence-absence heatmap of functional gene annotations of the vOTUs based on Pharokka-gv annotations. In the barplot above the heatmap are the number of predicted protein coding sequences per vOTU.

The majority of viral genes had an unknown function, however, based on the presence of lysis or integration and excision genes, two vOTUs were predicted to be lytic and three vOTUs predicted to be temperate (Fig. 4). No integrated prophages were predicted in the vOTUs or recovered from the MAGs. The number of predicted protein coding sequences (CDSs) in the vOTUs ranged from 4 to 120. No known virulence or antimicrobial resistance genes were identified in phage genomes, suggesting they did not contribute to potential SSA pathogenicity.

### Viral diversity as health biomarkers in Atlantic salmon

The Atlantic salmon microbiota have shown to experience an increase in bacterial diversity when suffering infection or if their system is disturbed by introduction of probiotic or synbiotic additives [16, 20, 66], suggesting the 16S rRNA gene profile of Atlantic salmon gut content could be used as a biomarker for disease [16]. Across all samples, we found that the disease state of the fish was reflected in the microbiota composition, with a few exceptions where sick fish microbiomes were dominated by SSM and fish labelled as healthy contained high abundance of SSA (Fig. 2C). The seven sick fish with unknown 16S rRNA gene based bacterial profiles all had viral communities that resembled viral profiles of the SSA dominated sick fish (*P* value = 0.85, Fig. 3A; supported by MAG read coverage, Fig. S3), and four of the seven microbiome samples specifically contained phages predicted to infect *Vibrionaceae* bacteria (Fig. 3D). Furthermore, the presence of vOTUs seemed to increase the sensitivity of predicting an early stage SSA colonisation, as the outlier samples in the SSM group (Fig. 3A), were from samples of phenotypically sick fish, which had a microbiota dominance of SSM > 90% according to the relative abundance of 16S rRNA gene presence (Fig. 3C, 2D).

### Metabolic niche of host bacteria as an indicator for associated phage community diversity

**Figure 5:**
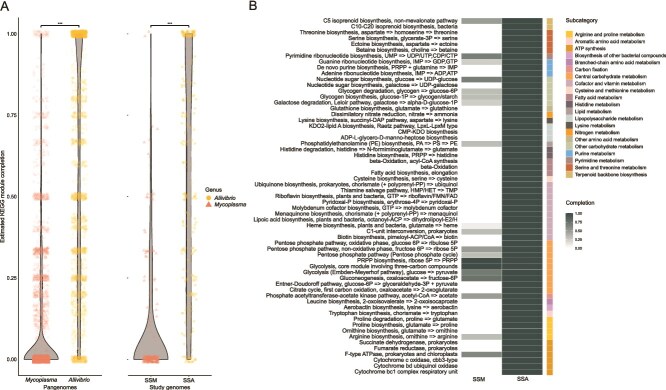
Estimated KEGG metabolic pathway completion with Wilcoxon rank sum test showing a significant difference between the two bacterial groups. *P* values are displayed accordingly: ^*^  *P* > .05, ^**^  *P* > .01, ^***^  *P* > .001. (A) Comparison of metabolic module completion between fish-associated *Mycoplasma* and *Aliivibrio* at the pangenome level (left), and between SSM and SSA MAGs (right). The MAGs exhibit similar completion patterns to their respective pangenomes. (B) Selecting for complete pathways, only six module subcategories are found in the SSM MAG compared to 21 in the SSA MAG.

A key component of phage diversity are the genetic properties of the bacterial host [67]. To explore potential drivers of the observed viral community composition, we investigated the metabolic niches of the two dominant bacterial groups by reconstructing and comparing their metabolic profiles. The metabolic niche refers to the genetic capacities that shape how microorganisms function within shared environments [68]. The genera *Aliivibrio* and *Mycoplasma* are phylogenetically distant, diverging at the kingdom level, and represent fundamentally different bacterial lineages with distinct genomic repertoire. Based on KEGG module completeness, we explored the metabolic capacity in both the SSA and SSM MAGs, and their associated fish-related pangenomes. Although the limited metabolic capabilities have been well documented across various species of *Mycoplasma* [66, 69, 70], the specific metabolic profile of SSM remains uncharacterised. Likewise, SSA represents an undescribed *Aliivibrio* species whose genomic and metabolic characteristics have not been explored. Analysis of the pangenome collections revealed a significant difference in metabolic capacity between the two groups. *Mycoplasma* genomes exhibited a low metabolic capacity, with an average KEGG module completion of just 9%, indicating limited independent functionality and a high host dependence. In contrast, *Aliivibrio* genomes showed a mean module completion of 56%, consistent with a metabolically independent and potentially opportunistic lifestyle (Fig. 5A). The two MAGs, SSM and SSA, mirrored these patterns, aligning closely with their respective genus-level profiles (Fig. 5A). To determine which functions contributed to these differences, we focused on pathways estimated to be complete in either MAG. KEGG subcategories classify modules into distinct areas of metabolism, such as amino acid biosynthesis or energy production [43]. Complete pathway modules in the SSM MAG were found in only 6 of 21 KEGG subcategories, whereas SSA had at least one complete module in every subcategory (Fig. 5B). Together with the significantly different richness of vOTUs related to SSM and SSA (Fig. 3A), metabolic capacity estimates may in part explain the relationship, or lack thereof, between viral communities in salmon dominated by either SSM or SSA.

Our metabolic niche analysis of the two bacterial groups revealed that SSA possessed significantly higher metabolic capacity, potentially explaining the enhanced viral richness observed in SSA dominated samples. To further investigate signatures of phage pressure on the SSA and SSM genomes, we examined their predicted phage defence mechanisms. Only a single defence mechanism was identified in the SSM MAG, belonging to the phage defence candidate PDC-S14. This mechanism is proposed to function via restriction-modification enzyme activity, exhibiting similarities with the GAO_29 defence system [71, 72]. The SSA MAG, in contrast, was predicted to encode 11 different defence mechanisms, ranging from nucleotide depletion, restriction-modification, abortive infection systems, the Lamassu family, and multiple phage defence candidate systems (Supplementary information).

## Discussion

We leveraged the low diversity microbiota ecosystem from a cohort of farmed Atlantic salmon [16], which exhibited clear disease-associated microbial shifts, to examine the viral communities associated with the two dominant bacteria in the salmon gut microbiome: *Aliivibrio* sp., SSA, and *Mycoplasma* sp., SSM. Disease in this salmon cohort was associated with a clear compositional shift where the resident gut symbiont SSM was replaced by the opportunistic coloniser SSA in response to ulcer-inducing *T. dicentrarchi* skin infection. The combination of elevated seawater temperatures and stress related to increased salinity may have compromised host barrier function and immune responses, as temperature-induced changes in the skin mucus and epithelial integrity can enhance pathogen binding [73], subsequently altering gut physiology in ways that favour opportunistic colonisers over resident symbionts [74]. Bacterial species within the *Aliivibrio* genus are all associated with marine animals, and with clear parallels to the *Mycoplasma* genus, they can act either as a mutualistic or antagonistic symbionts [75, 76]. Both bacterial species remain uncultured despite their importance in salmon health [10, 16], along with the majority of bacteria and their viruses across biomes [77]. SSM and similar strains of *Mycoplasma* have co-diverged with Atlantic salmon and are believed to contribute to host health through nutrient metabolism [10], whereas opportunistic colonisers such as SSA can disrupt this balance and cause dysbiosis even when they are not pathogenic [78]. The ratio of *Bacillota* (*Mycoplasma*) to *Pseudomonadota* (*Aliivibrio*) mirrors patterns observed in previous studies, where decreased *Bacillota*:*Pseudomonadota* ratios in gut microbiota have been linked to intestinal inflammation in both fish and other vertebrates [74, 79].

The substantial evolutionary divergence between these two dominant bacterial taxa, coupled with their distinct ecological strategies, revealed fundamental relationships between bacterial lifestyle and phage diversity. Specifically, we observed distinctive patterns of phage diversity associated with colonising and highly host-dependent bacteria. This phylogenetic separation, combined with the near-binary distribution of the two bacterial groups across samples, provided a framework for identifying virus-host associations through co-occurrence patterns, circumventing methodologies typically required in more diverse microbial communities [80]. The discrete phage populations uniquely associated with SSA and SSM further allowed us to infer bacterial community composition in seven samples with unresolved microbiota composition. These seven samples with unknown bacterial composition were selected to obtain even numbers of healthy and sick fish in the analysis and were expected to be dominated by SSA [16]. From the viral community composition, we predicted that the bacterial community of the sick fish to be dominated by SSA, supporting the findings that disease onset causes a switch in gut microbiota composition towards *Aliivibrio* [16]. Even when SSA had a relative abundance of less than 15%, the impact of its emerging colonisation was evident from the accompanying changes in phage community composition. Recent meta-analyses demonstrate that shifts in viral community composition and enrichment of specific viral taxa are consistent signatures of microbiome dysbiosis across diverse host systems [81]. These findings highlight, not only the potential of viral community composition to infer bacterial community dynamics, but also the emerging potential of phages as health biomarkers in stable versus disturbed microbiomes.

Phage-bacterial interactions represent a complex and dynamic evolutionary relationship, characterised by continuous genetic adaptations and counteradaptations. Although the average bacterial genome harbours approximately five defence systems, certain phyla, including *Mycoplasma*, have a reduced repertoire of genome encoded defence mechanisms [82]. Species of *Mycoplasma* are facultative or obligate intracellular and have reduced genomes [83, 84]. It has been argued that some bacterial endosymbionts are not infected by phages [85]. This is in accordance with our findings in salmon, as we did not find any correlation between vOTUs and samples with high SSM dominance, nor vOTUs in any samples predicted to infect a *Mycoplasma*. Accordingly, we were unable to recover vOTUs from many SSM-dominated samples, despite high bacterial densities. Whereas our current evidence did not indicate the presence of specific phages associated with SSM, other studies have identified phages infecting *Mycoplasma* species [86], and a recent genomic study found prophages across multiple *Mycoplasma* genomes [87]. Hence, even though we did not identify SSM-specific phages in the salmon gut microbiome, it is likely that phages are interacting with Mycoplasma in this environment, as also indicated by the presence of a phage defence system in the SSM genome.

**Figure 6:**
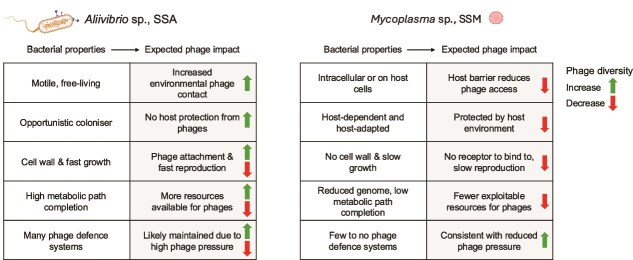
Summary figure of ecological drivers shaping the interaction between the two dominant bacteria, SSA (left) and SSM (right), and phage community. We propose that the fundamental differences of the two bacteria, ranging from interactions with animal host, to cellular structures, reproduction and metabolic capabilities, are mirrored in their susceptibility to phages and shape the diversity of their associated viral community. Icons created with BioRender.com.

In general, only a few phages have been reported to infect *Mollicutes* despite extensive efforts to identify such phages [86]. Furthermore, the intracellular niche of *Mycoplasma* species, might limit the encounter with mobile genetic elements and phages [88], highlighting the importance of bacterial niche ecology for the exposure to, and expected interaction with, phages [89]. To predict the ecological niche of bacteria that coexist within the same environment, one effective approach is to examine their metabolic capability [68]. The metabolic niche is defined by predicted metabolic capabilities and provides insight into habitat preferences, including host dependence, as well as ecological dynamics, such as distinguishing generalists from specialists. The distinct metabolic potentials of SSM and SSA, combined with their potential role in the salmon gut [10, 18], emphasise their unique ecological niches (Fig. 6). Despite the remarkable difference and impact of metabolic niches across bacteria [90], how and whether these niches relate to the associated phage communities has received limited attention. One potential explanation for the contrasting phage diversities we observed between SSA and SSM lies in their differing metabolic niches. SSM, characterised by a reduced genome, exhibits a limited metabolic capacity, in clear contrast to the SSA, which possesses a more expansive genome with a diverse array of complete metabolic pathways. We hypothesise that the metabolic capacity of bacteria not only reflects their evolutionary history, ecological strategies, and environmental adaptations, but is also invariably linked to phage interactions and viral community diversity. Studies on the impact of multiple phages infecting the same host, have identified phage-specific metabolic changes and requirements, including increased pyrimidine and amino acid metabolism [91–93]. Pyrimidine nucleotides are essential for phage DNA replication, whereas compounds such as putrescine, a metabolite associated with the amino acid arginine and proline metabolism, contributes to the efficiency of genome packaging and translation [94], proposed to perform a similar role in phage replication [93]. We compared these phage-relevant metabolic pathways between SSA and SSM. SSA possessed complete pathways for pyrimidine and purine biosynthesis, and arginine/proline metabolism, whereas these pathways were either incomplete or absent in SSM. The metabolic pathways influenced by phage replication vary widely depending on infection stage (early, middle, or late genes) and phage type, as different phage strategies require different resources at different times [91–93, 95, 96]. For instance, one study found that arginine and purine biosynthesis were significantly upregulated during early infection but downregulated in late stages, with patterns varying across different phages [92]. Therefore, a metabolically limited host may only support phages whose resource requirements align with its available pathways, constraining phage community diversity. This metabolic-phage relationship is consistent with the phage communities we recovered: diverse phage communities in SSA-dominated samples and limited phage recovery in SSM-dominated samples. The metabolic independence that makes SSA an efficient gut coloniser [97], enabling fast growth and adaptation, may therefore come with the trade-off of increased phage pressure due to its capacity to support diverse phage types.

To date, the described phage community associated with any *Aliivibrio* species is very limited, with only a few phages described, which are all connected to *A. fisherii* [11, 98]. Given the phylogenetic similarity between *Aliivibrio* and the *Vibrio* genus, one would anticipate comparable phage community dynamics in the two genera. The *Vibrio* genus has a well-documented and extensive repertoire of phage interactions that play significant roles across *Vibrio* species [98–100]. The SSA were associated with multiple vOTUs and encoded an extensive collection of defence mechanisms indicating high phage pressure. Of the 22 vOTUs, both lytic or temperate infection strategies were observed, suggesting interactions that allowed co-existence of SSA with their phages. The concrete mechanisms and dynamics of bacterial coexistence with diverse phage communities remain intricate but deserve more attention as this field evolves.

We identified conservative estimates of inherent viral communities of two phylogenetically and ecologically distinct bacteria in the gut environment of genetically similar salmon reared under identical conditions. Our findings highlight the species-driven phage diversity in this system and offer broader perspectives on factors influencing phage community composition. We demonstrate how opportunistic colonisation of a bacteria, during stress or disease conditions of the salmon host, significantly alters the viral community compared to stable setting microbiota. These insights advance our understanding of phage-host coexistence and the potential application of phages against bacteria within specific ecological niches. Exploring the relationship between bacterial metabolic niches and phage diversity holds great potential to increase our understanding of these cellular parasites and their impact on bacterial ecology and evolution.

## Supplementary Material

supplementary_materials_wraf272

## Data Availability

The sequencing data generated for the study is available in NCBI Sequence Read Archive (SRA) with the project number PRJNA1260422. Contigs from both co- and individual sample assembly, including viral contigs, and MAGs are available on figshare https://doi.org/10.6084/m9.figshare.29986732.

## Code availability

The code used for this study is available at: https://github.com/CarolineSophieWH/phage_diversity_atlantic_salmon2025.
