# Supplementary material for: Ecological strategies of bacteria shape inherent phage diversity in Atlantic salmon gut microbiomes
Source: ISME J. 2025 Dec 8;19(1):wraf272. doi: 10.1093/ismejo/wraf272 (PMC12753311; doi:10.1093/ismejo/wraf272)
Supplement: supplementary_materials_wraf272 [file supplementary_materials_wraf272.zip › Supplementary_Figures.pdf]

Supplementary Figures

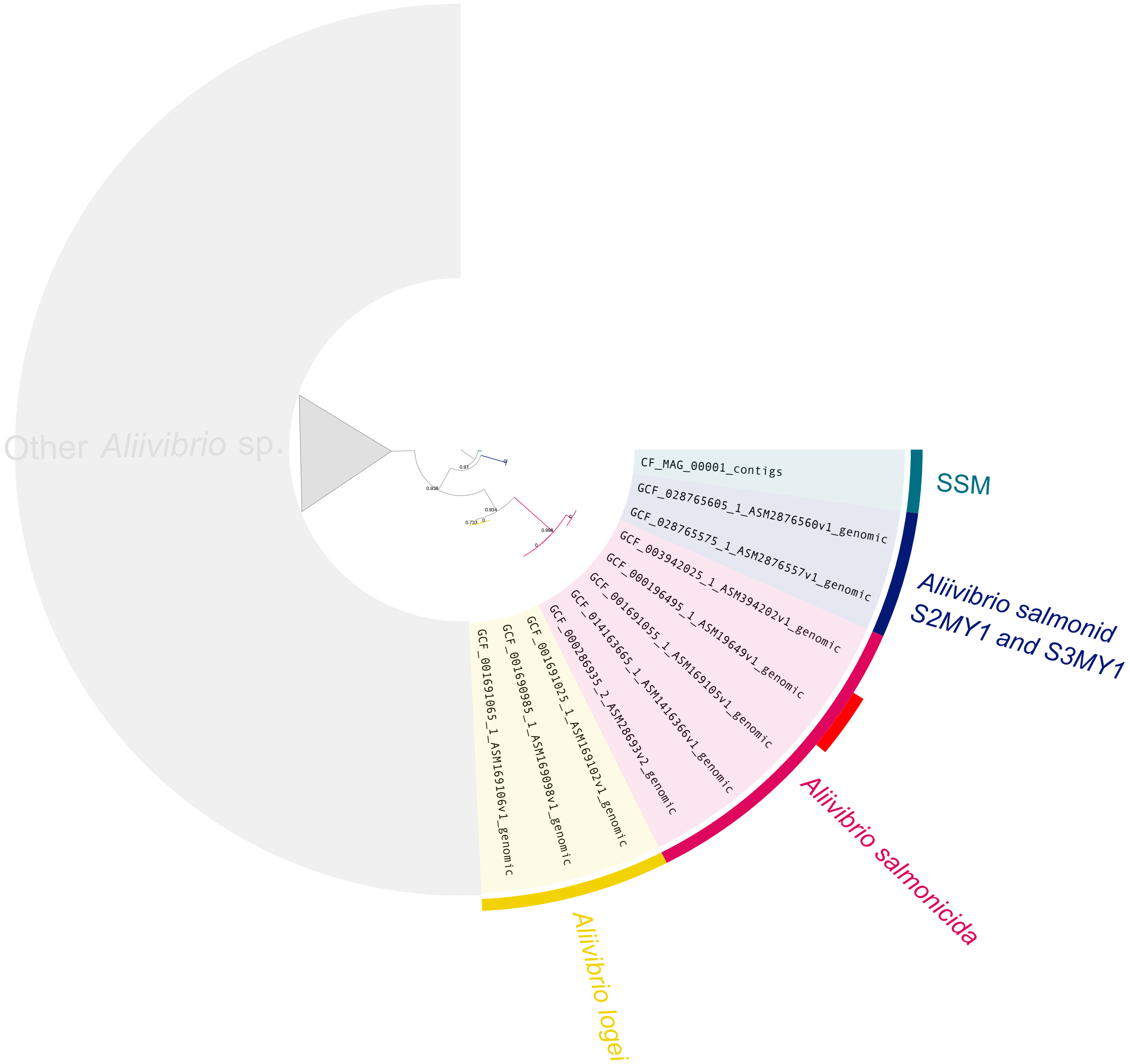

Figure S1. Phylogenomics of SSA and all publicly available *Aliivibrio* sp. genomes. The branch clustering with the MAG retrieved from this study is highlighted with the closest species from the isolate of *Aliivibrio* sp. S2MY1 and S3MY1 from a Tasmanian salmon farm, *A. salmonicida* and *A. logei*.

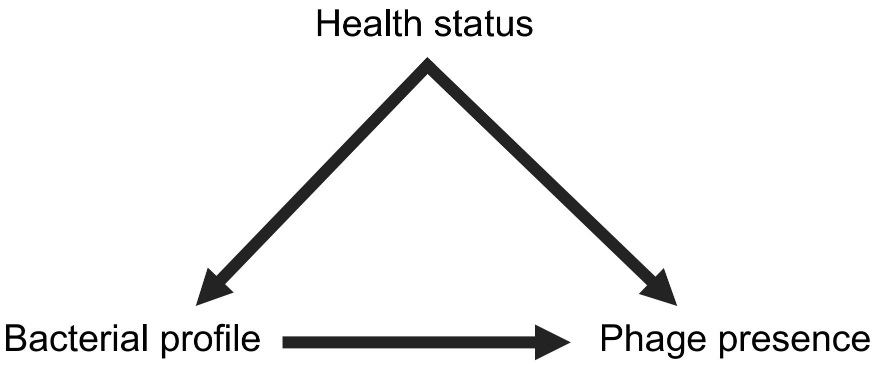

Figure S2. Directed acyclic graph (DAG) describing the assumed causal relationships between the variables.

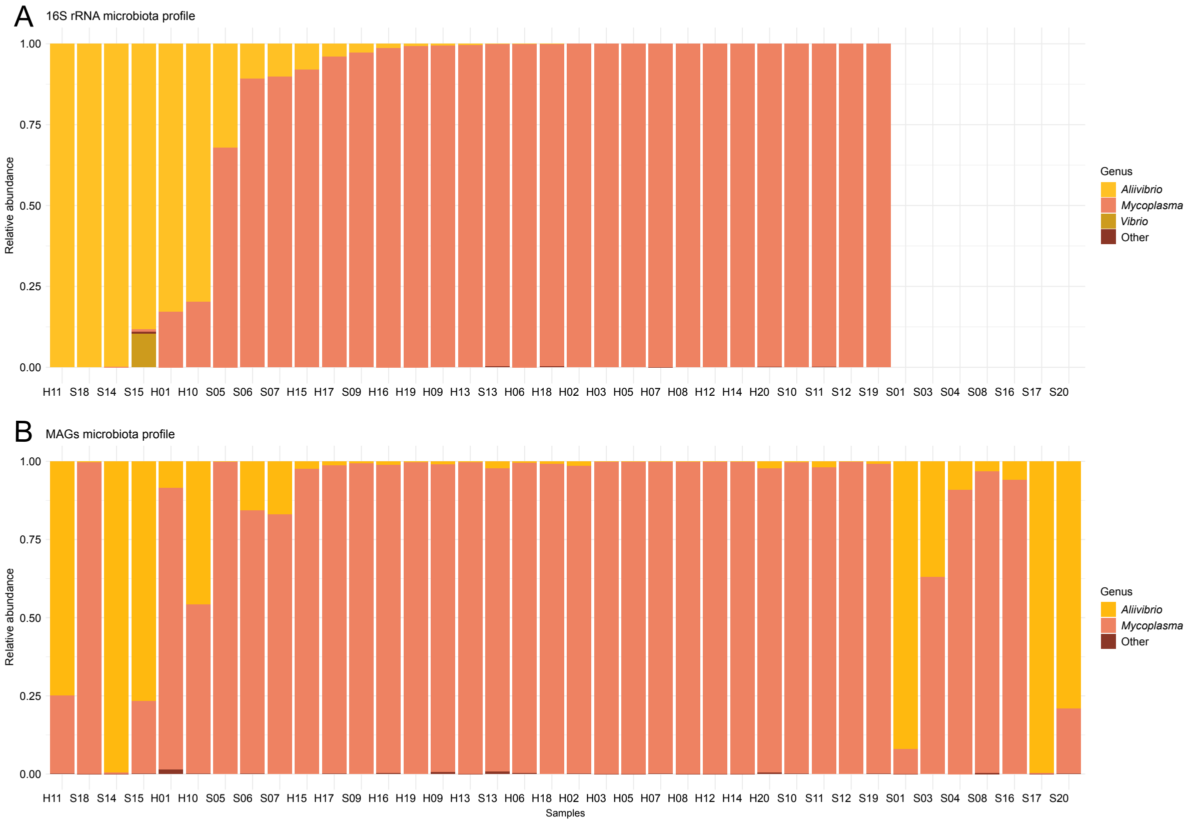

Figure S3. Relative abundance bar plots of normalised read coverage across samples from both 16S rRNA gene amplicon and viral-enriched metagenome data. A) Reads across the 31 samples from which 16S data was retrieved, sorted based on *Aliivibrio* read percentage. The seven samples missing on the right side represent the additional samples included to ensure equal numbers of sick and healthy fish phenotypes. B) SSM, SSA and *Pseudomonas* MAG reads from all 38 samples, sorted according to the 16S rRNA microbiota profile. Unbinned reads were not included. The seven samples lacking 16S rRNA data display similar composition to the SSA-dominant group and match the overall microbiota profile characteristic of the sick fish phenotype.

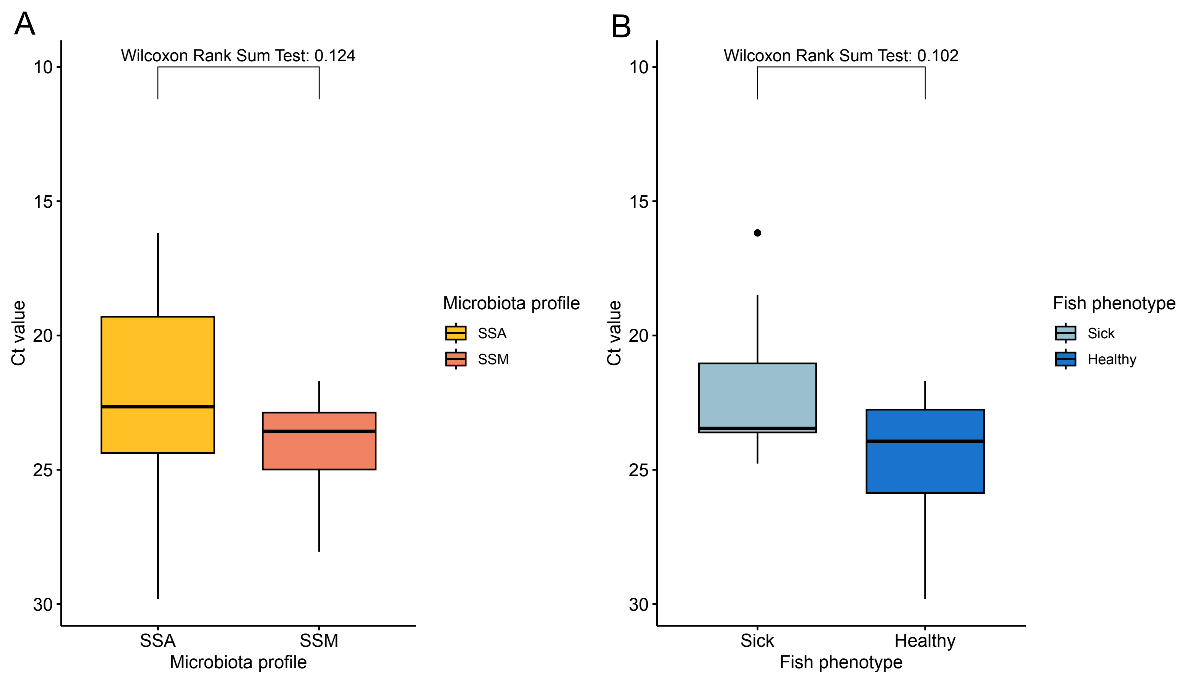

Figure S4. Cycle threshold (Ct) values from a qPCR assay across the 31 samples with 16S rRNA data. A) Ct values based with samples grouped by microbiota profile, SSM sp. (n=21) and SSA sp. (n=9). B) Ct values for samples grouped by disease phenotype, sick fish (n=12) and healthy fish (n=19).

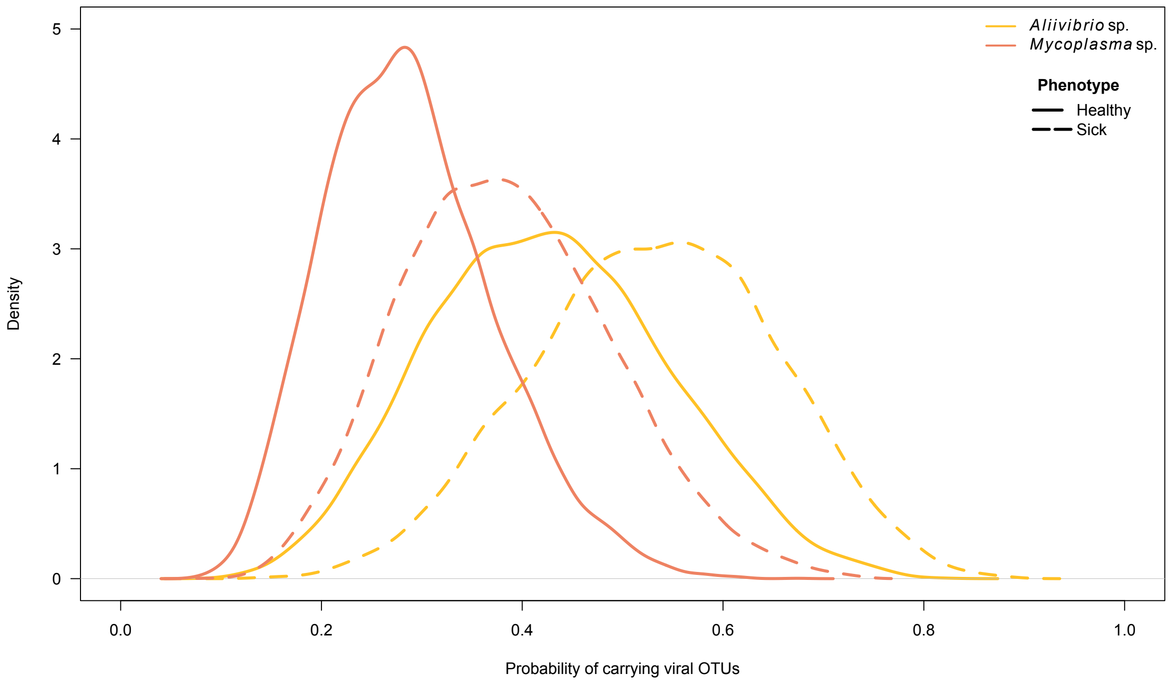

Figure S5. Posterior distribution of the probability of detecting viral OTUs in samples, stratified by health status and bacterial profile.

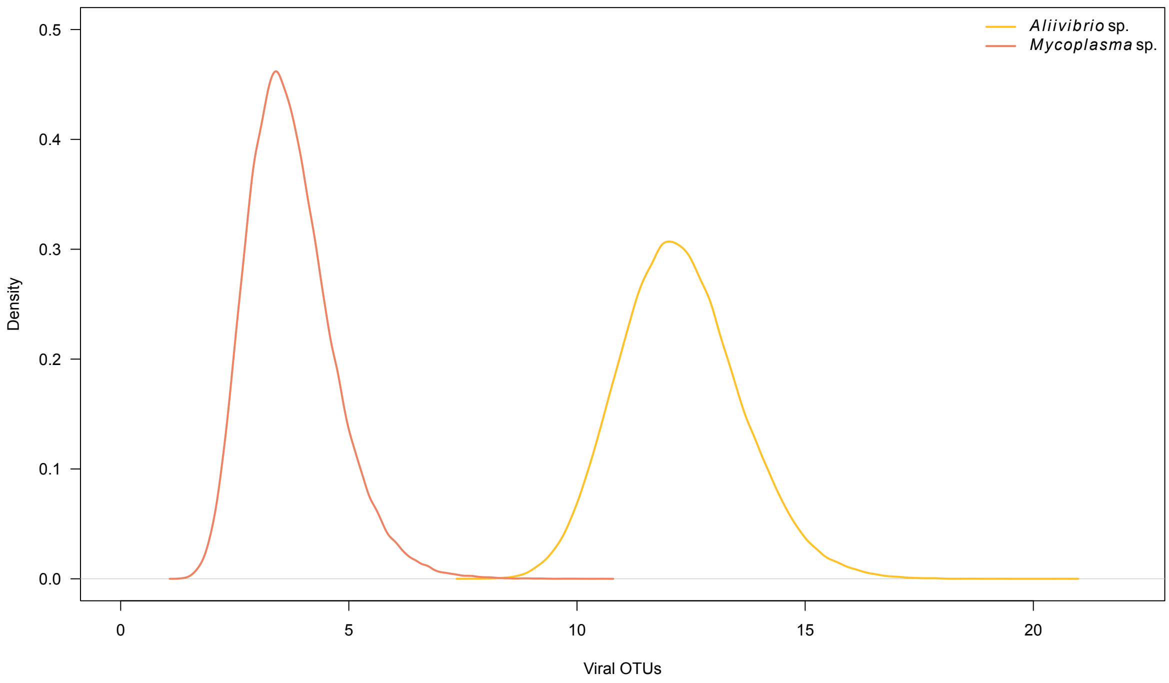

Figure S6. Posterior density for the number of viral OTUs detected per fish for the two categories of bacterial profiles.
